# Longitudinal associations between psychotic experiences and disordered eating behaviours in adolescence: a UK population-based study

**DOI:** 10.1016/S2352-4642(18)30180-9

**Published:** 2018-08

**Authors:** Francesca Solmi, Daniela Melamed, Glyn Lewis, James B Kirkbride

**Affiliations:** a Division of Psychiatry, University College London, London, UK

## Abstract

**Background:**

Psychotic experiences might represent non-specific markers of poor mental health in adolescence. However, only a few predominantly cross-sectional studies have tested their association with disordered eating behaviours in adolescent and adult populations. The aim of this study was to explore the association between psychotic experiences at age 13 years, and disordered eating behaviours and body-mass index (BMI) at age 18 years.

**Methods:**

We used data from the Avon Longitudinal Study of Parents and Children, a longitudinal birth cohort based in Avon (England, UK) including mothers with an expected delivery date between April 1, 1991, and Dec 31, 1992, and their children. Psychotic experiences (such as delusions and hallucinations) and BMI were measured at clinical assessments when children were nearly aged 13 years, and data on disordered eating behaviours (ie, presence of binge eating, purging, fasting, or excessive exercise for weight loss; any of these behaviours [included to increase statistical power]; and number of behaviours [included to investigate severity]) were obtained via a postal questionnaire that used adapted questions from the Youth Risk Behaviour Surveillance System questionnaire at approximately age 18 years. For each outcome, we ran a univariable model and four multivariable models (logistic, linear [for BMI], or negative binomial [for the number of behaviours] regression), progressively adjusting for child and maternal sociodemographic, physical, and mental health characteristics (including child's sex, and maternal age, marital status, and highest academic qualification); autistic traits at age 7 years (measured with the Social and Communication Disorder Checklist); baseline BMI at age 13 years, and depressive symptoms at baseline (ie, at age 13 years when psychotic experiences were measured: childs' symptoms measured with the Moods and feelings Questionnaire, and maternal symptoms measured at 32 weeks' gestation with the Edinburgh Postnatal Depression Scale). We imputed missing outcome and covariate data.

**Findings:**

Our sample included 6361 children, of whom 734 (12%) reported psychotic experiences at age 13 years. In univariable models, psychotic experiences were associated with greater odds of reporting any disordered eating behaviours (odds ratio [OR] 1·92, 95% CI 1·46–2·52; p<0·0001), and more severe symptoms (as measured by the number of disordered eating behaviours: 0·58, 0·32–0·84; p<0·0001) at age 18 years. These associations were slightly attenuated by adjustment for maternal and child characteristics (any disordered eating behaviours OR 1·82, 95% CI 1·35–2·44, p<0·0001; number of disordered eating behaviours 0·49, 95% CI 0·23–0·75, p<0·00001), autistic traits at age 7 years (any disordered eating behaviours OR 1·80, 95% CI 1·34–2·41, p<0·0001; number of disordered eating behaviours 0·48, 95% CI 0·22–0·74, p<0·00001), and BMI (any disordered-eating behaviours OR 1·83, 95% CI 1·36–2·46, p<0·0001; number of disordered-eating behaviours 0·32, 95% CI 0·06–0·57, p<0·00001) Adjusting for baseline depressive symptoms attenuated, but not removed, these associations (any disordered eating OR 1·50, 95% CI 1·10–2·03, p=0·010; more severe symptoms 0·32, 0·06–0·57, p=0·017). Psychotic experiences were also associated with greater binge eating, purging, and fasting behaviours, although some associations weakened after controlling for depressive symptoms. We noted no associations between psychotic experiences and excessive exercise or BMI in any of the models.

**Interpretation:**

Our findings suggested that psychotic experiences are markers of increased risk for several disordered eating behaviours in late adolescence, possibly by indicating more severe psychopathology in early adolescence. More research investigating shared risk factors for psychotic experiences and eating disorders is warranted to elucidate shared and specific causal pathways.

**Funding:**

Wellcome Trust, the Royal Society, University College London Hospitals National Institute for Health Research Biomedical Research Centre, UK Medical Research Council, and the University of Bristol.

## Introduction

Psychotic experiences, such as delusions or hallucinations (core symptoms of schizophrenia), are common in the general population, especially in childhood and adolescence.^1^ Findings from a meta-analysis^1^ suggested that the median prevalence of psychotic experiences in late childhood and early adolescence (age 9–12 years) is 17%, decreasing to 7·5% in late adolescence^1^ and reaching a prevalence of around 5% in adulthood.^2^ Traditionally, psychotic experiences have been predominantly conceptualised as prodromal expressions of psychotic disorders. However, an increasing number of studies have shown that they are also longitudinally associated with a range of adverse psychopathological outcomes—including mood disorders,^3^ substance use,^3^ post-traumatic stress disorder,^5^ and suicide attempts^5^, ^6^—suggesting that they could represent early transdiagnostic, non-specific markers of mental health disorders.^7^

**Research in context**

**Evidence before this study**

We searched MEDLINE, PubMed, and Google Scholar for articles published between 2003–18, on the comorbidity of psychotic symptoms (search terms “psychotic symptoms” and “psychosis”) and eating disorders (“eating disorders”, “anorexia”, “bulimia”, and “binge eating”). We noted three studies that had investigated this association. These studies suggested that both adolescents and adults with psychotic symptoms were more likely to experience eating-disorder behaviours (and vice-versa) or have a comorbid diagnosis of an eating disorder over the course of their lifetime. One study showed that adolescents and adults with psychotic symptoms were more likely to develop bulimia nervosa. To date, studies have predominantly investigated this association cross-sectionally, or relied on study participant recall of onset of symptoms. From these approaches it is difficult to establish temporality of association—a precondition for causal inference—and exclude recall bias. Finally, most existing studies investigated eating-disorder diagnoses, but not eating disorder behaviours, which might be more common in the general population.

**Added value of this study**

To our knowledge, ours is the first study to investigate the association of psychotic experiences and disordered-eating behaviours longitudinally in a large sample of English adolescents. We noted evidence that children with psychotic experiences in early adolescence had increased odds of reporting disordered eating in late adolescence and having more severe presentations, denoted by a greater number of disordered-eating behaviours. Compared with previous studies, our approach has the advantage of being able to exclude recall bias, limit the potential for reverse causation, and control for several potential child and familial confounding factors.

**Implications of all the available evidence**

Despite little research attention, evidence suggests that psychotic symptoms and eating disorders can co-occur, as observed in clinical reports and highlighted in the epidemiological literature. Psychotic experiences in adolescence have been previously shown to be a non-specific early marker of adverse psychopathologies, which we extend to include eating disorders. Future studies should investigate the presence of common and specific risk factors which may underpin their causes. The longitudinal associations we observed between psychotic experiences and disordered eating could indicate the presence of shared aetiological mechanisms. Furthermore, the greater prevalence of bingeing, fasting, and purging behaviours among children with psychotic experiences could partly explain the higher rates of metabolic disorders in individuals with psychotic illnesses or indicate shared metabolic risk factors, although further research in this area is warranted.

Eating disorders (anorexia nervosa, bulimia nervosa, and binge-eating disorder) are comorbid with mood disorders, substance use, post-traumatic stress disorder, and suicide attempts;^8^, ^9^, ^10^ nevertheless, their association with psychotic experiences has received little attention in the epidemiological literature. To our knowledge, only two studies have investigated this association in the general population, both of which were based on adult samples.^4^, ^11^ One cross-sectional survey showed that participants who screened positive for eating disorders using the SCOFF five-item screening questionnaire for eating disorders had increased odds of reporting psychotic experiences and hypomania, and that those reporting loss of control when eating (which is a symptom of binge eating) had greater odds of experiencing auditory hallucinations and other psychotic experiences.^4^ In line with this finding, results from a multisite study showed that psychotic symptoms were associated with twice the odds of subsequently experiencing bulimia nervosa, an eating disorder characterised by recurrent episodes of binge eating and purging (ie, self-induced vomiting).^11^ Finally, findings from another study showed that adolescents with clinical diagnoses of anorexia and bulimia nervosa were more likely to have psychotic experiences than general population controls.^12^ These findings are largely similar to those from clinical studies showing a comorbidity between psychotic illnesses, including schizophrenia and bipolar disorder, and disordered eating behaviours and diagnoses of eating disorders.^13^, ^14^

Although evidence suggests that psychotic experiences and eating disorders can co-occur,^3^, ^11^, ^12^ several methodological limitations of these studies preclude causal inferences being made from these associations. For instance, the cross-sectional^3^, ^12^ and retrospective designs^11^ of these studies prevented the investigation of temporal associations. Additionally, because of the under-representation of eating disorders in clinical settings, the use of clinical samples^12^ could also have resulted in selection bias and overlooked psychopathological presentations more commonly observed in the general population. Longitudinal research on the association between psychotic experiences and disordered eating is therefore needed to elucidate the temporality of these associations and their specificity to individual disordered-eating behaviours, which are both necessary preconditions for causal inference.

We investigated whether psychotic experiences at age 13 years were prospectively associated with disordered-eating behaviours at age 18 years in a longitudinal, general population sample of UK adolescents. Based on previous evidence we hypothesised that psychotic experiences would be associated with greater disordered eating behaviours and body-mass index (BMI).

## Methods

### Data sample

We used data from the Avon Longitudinal Study of Parents and Children (ALSPAC), a longitudinal birth cohort based in Avon (England, UK). All mothers in this region with an expected delivery date between April 1, 1991, and Dec 31, 1992 were eligible and invited to participate in the study. 14 541 pregnant women were recruited for the study and resulted in 13 988 live births at 1 year. Information on these children and their mothers' health and life circumstances has since been collected through clinical visits and self-reported questionnaires. More details on the ALSPAC sample and measures collected have been previously published.^15^ The local research ethics committees at the University of Bristol (Bristol, England, UK) and the ALSPAC Law and Ethics Committee provided ethical approval for this study. Parents provided written consent for their children's participation in the study. The ALSPAC study website contains details of all the data that are available through a fully searchable dictionary on the study website.

### Exposure

Data on psychotic experiences were collected at clinic assessments done when children were nearly 13 years old using the psychotic-like symptoms interview, a semi-structured interview consisting of 12 questions adapted from the Diagnostic Interview Schedule for Children^16^ and the Schedules for Clinical Assessment in Neuropsychiatry^17^ assessing the presence of hallucinations, delusions, and thought interference. From the total interview score, a binary variable was created to indicate the absence of psychotic experiences or the presence of suspected or definite psychotic symptoms. Children were defined as having any suspected or definite psychotic experiences if they reported one or more such experiences that could not be attributable to an absence of sleep or fever. This measure has been previously validated^18^ and used extensively in general population studies,^19^, ^20^, ^21^, ^22^ thus ensuring comparability of our findings.

### Outcomes

Information on disordered-eating behaviours was collected via postal questionnaires at age approximately 18 years using adapted questions of the Youth Risk Behaviour Surveillance System questionnaire.^23^ We derived four binary variables indicating the presence or absence of binge eating, purging, excessive exercise, and fasting in the previous 12 months. Binge eating was defined as having ever eaten large amounts of food in short periods of time with usually a sense of loss of control during these episodes. Children were considered as purging if they had ever had self-induced vomiting or had used laxatives for weight loss, and as fasting if they had ever fasted for a whole day to lose weight. Excessive exercise was defined as exercising frequently for weight loss even when sick, or feeling guilty when not exercising, as these dimensions (as opposed to objective amounts of exercise undertaken, for example) have been shown to underlie eating-disorder psychopathology.^24^ A more detailed description of the original questions included in the age 18 years questionnaire and how we derived our outcome variables is in the appendix. Because our individual outcomes were rare, we also investigated the presence of any disordered eating behaviours to increase the statistical power, and the number of disordered eating behaviours (from none to four) to explore the presence of an association between psychotic experiences and more severe presentations of eating disorders, defined as a greater number of disordered-eating behaviours. A continuous standardised measure of BMI was derived from objective measures of height and weight obtained at clinic visits that took place when the child was approximately age 18 years.

### Confounders

Analyses were adjusted for several child and maternal characteristics that we hypothesised could confound the association between psychotic experiences and eating-disorder behaviours, based on findings from previous studies (appendix). We modelled our assumptions using a direct acyclical graph^25^ and the web programme DAGgitty (appendix).^26^ We included child's sex (male or female), autistic traits at age 7 years, and BMI and depressive symptoms at age 13 years. To measure autistic traits in the children, we used the Social and Communication Disorder Checklist (SCDC), a parent-reported 12-item scale that measures deficits in verbal and non-verbal social communication, typically observed in autism spectrum disorders.^27^ Total SCDC scores ranged from 0 to 24, with higher scores denoting greater social communication difficulties. Depressive symptoms were measured using the short Moods and Feelings Questionnaire, a self-reported 13-item questionnaire with scores ranging from 0 to 26, with greater scores indicating more severe depressive symptoms.^28^ We further adjusted for the maternal characteristics of age, marital status (single, married, divorced, separated, or widowed), and highest academic qualification (up to secondary school, or university degree or higher), all measured at birth of the child. We also included a continuous measure of maternal depressive symptoms at 32 weeks of gestation using the Edinburgh Postnatal Depression Scale, a ten-item questionnaire from which we derived a continuous measure ranging from 0 to 30; this scale has been previously validated and extensively used in epidemiological research.^29^ Finally, we adjusted for baseline BMI at age 13 years, which was measured using standardised age and gender-specific *Z* scores using the updated Stata package zanthro,^30^ as recommended in previous studies.^31^, ^32^

### Statistical analysis

We used frequencies (with percentages), means (with SDs), and medians (with IQRs) to describe the distribution of confounders and outcomes in relation to the exposure. To investigate the association between psychotic experiences and disordered eating behaviours (ie, binge eating, purging, fasting, excessive exercise, with any behaviours), we used a univariable (crude model) and four multivariable logistic regressions models progressively adjusting for maternal and child confounders. In adjusted model 1, we controlled for children's sex, and maternal characteristics of age, social class, marital status, and depressive symptoms. In adjusted model 2, we further controlled for autistic traits; in adjusted model 3 we subsequently included BMI at age 13 years; and in adjusted model 4, child's depressive symptoms. To model the associations between psychotic experiences and BMI we used linear regressions, whereas for the number of disordered-eating behaviours we used negative binomial regression models, as these were best suited to model count data in the presence of overdispersion of data (ie, when the SD is greater than the mean), following the same model-building approach described above.

We explored the effect of missing data using multiple imputation by chained equations.^33^ We imputed missing covariate and outcome data using linear, logistic, and multinomial logistic regression models (as appropriate) using the MI impute chained Stata command and 50 imputed dataset, using Rubin's rules^34^ to estimate combined effect sizes. In our imputation models we included all variables used in our main model and the number of auxiliary variables putatively associated with the imputed variables and outcome missingness, as recommended in a previous study.^35^ These variables were low birthweight (<2500 g or ≥2500 g); intelligence quotient at age 7 years; BMI and disordered behaviours at age 16 years; a continuous measure of depressive symptoms, measured with the short Moods and Feelings Questionnaire at age 16 and 18 years;^36^, ^37^ polygenic risk scores for schizophrenia;^38^ a continuous measure of negative symptoms at age 16 years measured with ten items from the Community Assessment of Psychic Experiences; and sexual orientation as this has been shown to be associated with disordered eating in this sample^39^ as well as missingness.

Because the results of the imputed models did not differ qualitatively from those of complete case analyses (ie, point estimates were similar and 95% CI overlapped), in the results section we present models based on a sample of participants with complete exposure data and imputed confounders and outcome (sample A) and discuss any observed differences in results compared with complete cases analyses (sample B). We also compared these results with a set of analyses done on a sample of children with complete exposure and outcome data, but also missing, and hence imputed, confounders (sample C). All analyses were done using Stata v.13.

### Role of the funding source

The funder of the study had no role in study design, data collection, data analysis, data interpretation, or writing of the report. The corresponding author had full access to all the data in the study and had final responsibility for the decision to submit for publication.

## Results

We obtained data on psychotic experiences at age 13 years for 6361 children (table 1). Of these, most were girls and had mothers educated up to secondary school who were married and had non-manual occupation. A greater proportion of children with psychotic experiences at 13 years were girls, reported greater depressive symptoms and autistic traits, had younger mothers who were single, separated, or widowed, and had lower levels of education and higher depressive symptoms.

**Table 1:** Descriptive statistics of psychotic experiences at age 13 years with confounding and outcome variables

		Total*	**Psychotic experiences at 13 years**	
			No	Yes
Total		6361 (100%)	5627 (89%)	734 (12%)
Sex				
	Male	3131 (49%)	2794 (50%)	337 (46%)
	Female	3230 (51%)	2833 (50%)	397(54%)
Maternal marital status				
	Single (never married)	863 (14%)	733 (13%)	130 (19%)
	Married	5046 (81%)	4523 (82%)	523 (74%)
	Widowed, divorced, or separated	302 (5%)	251 (5%)	51 (7%)
Maternal highest academic qualification				
	Compulsory education	5133 (84%)	4518 (83%)	615 (87%)
	Degree or higher	1005 (16%)	914 (17%)	91 (13%)
Binge eating at 18 years				
	No	2474 (96%)	2236 (96%)	238 (92%)
	Yes	107 (4%)	87 (4%)	20 (8%)
Purging at 18 years				
	No	2408 (94%)	2177 (94%)	231 (90%)
	Yes	156 (6%)	130 (6%)	26 (10%)
Fasting at 18 years				
	No	2297 (89%)	2089 (90%)	208 (80%)
	Yes	291 (11%)	240 (10%)	51 (20%)
Excessive exercise at 18 years				
	No	2472 (96%)	2220 (96%)	252 (97%)
	Yes	101 (4%)	93 (4%)	8 (3%)
Any disordered eating at 18 years				
	No	2079 (82%)	1899 (83%)	180 (71%)
	Yes	453 (18%)	380 (17%)	73 (29%)
Maternal age		29·1 (4·6)	29·2 (4·5)	28·5 (4·8)
Maternal depressive symptoms		6·4 (4·6)	6·3 (4·5)	7·4 (4·8)
Child's BMI (*Z* scores)				
	At 13 years	0·3 (1·1)	0·3 (1·1)	0·3 (1·1)
	At 18 years	–0·3 (1·0)	–0·3 (1·0)	–0·2 (0·9)
Child's depressive symptoms at age 13 years		3 (1–5)	3 (1–5)	6 (3–10)
Child's autistic traits at age 7 years		2 (0–4)	1 (0–4)	2 (0–4)
Number of disordered-eating behaviours		6 (3–9)	6 (3–9)	7 (4–11)

Data are n (%), mean (SD), or median (IQR). BMI=body-mass index.

* Distribution (ie, numbers and proportions) of confounding and outcome variables refers to the sample of children with available exposure data (our analytical sample) shown in the first row of the table.

Among children with complete exposure data, around 30% did not have any outcome data at age 18 years (appendix). Children who were male, with greater autistic traits at age 7 years (and who had a mother who had only completed secondary education, was single, younger, and had greater depressive symptoms), had greater odds of not having outcome data available at age 18 years.

With the exception of excessive exercise all disordered eating behaviours were more prevalent among children with psychotic experiences at age 13 years. Overall, around a third of children with psychotic experiences reported any disordered eating behaviours, compared with 17% of children without psychotic experiences (table 1). Children with psychotic experiences also reported more disordered eating behaviours than those without psychotic experiences (median 7 [IQR 4–11] *vs* 6 [IQR 3–9]).

Children who reported psychotic experiences at 13 years had an increased risk of having any disordered eating behaviours at age 18 years in crude models (table 2) and in models adjusting for child and maternal confounders, autistic traits, BMI at 13 years, and depressive symptoms (table 2). Psychotic experiences at age 13 years were also associated with greater number of disordered eating behaviours in the crude model and in models adjusting for child and maternal confounders including depressive symptoms at baseline (ie, at age 13 years when psychotic experiences were measured).

**Table 2:** Logistic, linear (for BMI), and negative binomial (for number of disordered eating behaviours) regression models

		**Complete exposure, imputed confounders and outcome (sample A) Odds ratio (95%CI)**	**Complete cases (sample B) Odds ratio (95%CI)**
Binge eating		n=6361	n=1940
	Crude model	2·23 (1·36–3·69), p=0·0018	1·79 (0·96–3·30), p=0·062
	Adjusted model 1*	2·01 (1·20–3.37), p=0·0082	1·57 (0·84–2·95), p=0·16
	Adjusted model 2†	1·99 (1·18–3.33), p=0.0097	1·57 (0·83–2·94), p=0·16
	Adjusted model 3‡	2·04 (1·21–3·43), p=0·0081	1·60 (0·85–3·01), p=0·14
	Adjusted model 4§	1·49 (0·88–2·54), p=0·14	1·27 (0·66–2·45), p=0·48
Purging		n=6361	n=1926
	Crude model	1·88 (1·24–2·84), p=0·0031	1·99 (1·18–3·35), p=0·0089
	Adjusted model 1*	1·74 (1·13–2·68), p=0·013	1·85 (1·08–3·15), p=0·025
	Adjusted model 2†	1·73 (1·12–2·67), p=0·013	1·85 (1·08–3·15), p=0·025
	Adjusted model 3‡	1·76 (1·14–2·72), p=0·011	1·85 (1·08–3·16), p=0·025
	Adjusted model 4§	1·51 (0·96–2·36), p=0·071	1·85 (1·05–3·23), p=0·032
Excessive exercise		n=6361	n=1929
	Crude model	0·84 (0·40–1·78), p=0·65	0·53 (0·19–1·47), p=0·22
	Adjusted model 1*	0·79 (0·37–1·71), p=0·55	0·50 (0·18–1·39), p=0·18
	Adjusted model 2†	0·79 (0·37–1·71), p=0·55	0·49 (0·18–1·38), p=0·18
	Adjusted model 3‡	0·80 (0·37–1·73), p=0·57	0·49 (0·18–1·39), p=0·18
	Adjusted model 4§	0·71 (0·32–1·57), p=0·39	0·46 (0·16–1·32), p=0·15
Fasting		n=6361	n=1943
	Crude model	2·25 (1·64–3·09), p<0·0001	2·33 (1·58–3·44), p<0·0001
	Adjusted model 1*	2·09 (1·49–2·94), p<0·0001	2·26 (1·51–3·40), p<0·0001
	Adjusted model 2†	2·05 (1·46–2·89), p<0·0001	2·25 (1·50–3·38), p<0·0001
	Adjusted model 3‡	2·10 (1·49–2·96), p<0·0001	2·25 (1·50–3·39), p<0·0001
	Adjusted model 4§	1·65 (1·15–2·38), p<0·0001	1·94 (1·26–2·97), p=0·0025
Any disordered eating behaviour		n=6361	n=1898
	Crude model	1·92 (1·46–2·52), p<0·0001	2·06 (1·41–2·90), p<0·0001
	Adjusted model 1*	1·82 (1·35–2·44), p<0·0001	1·97 (1·37–2·83), p<0·0001
	Adjusted model 2†	1·80 (1·34–2·41), p<0·0001	1·97 (1·37–2·83), p<0·0001
	Adjusted model 3‡	1·83 (1·36–2·46), p<0·0001	1·98 (1·37–2·87), p<0·0001
	Adjusted model 4§	1·50 (1·10–2·03), p=0·010	1·70 (1·16–2·49), p=0·0070
Number of disordered-eating behaviours¶		n=6361	n=1898
	Crude model	0·58 (0·32–0·84), p<0·0001	0·54 (0·19–0·88), p=0·0021
	Adjusted model 1*	0·49 (0·23–0·75), p<0·0001	0·46 (0·13–0·79), p=0·0058
	Adjusted model 2†	0·48 (0·22–0·74), p<0·0001	0·46 (0·14–0·79), p=0·0054
	Adjusted model 3‡	0·49 (0·24–0·75), p<0·0001	0·48 (0·16–0·80), p=0·0036
	Adjusted model 4§	0·32 (0·06–0·57), p=0·017	0·38 (0·05–0·71), p=0·024
BMI¶^e^		n=6361	n=2957
	Crude model	0·026 (–0·06–0.11), p=0·54	0·016 (–0·09–0·12), p=0·77
	Adjusted model 1*	0·006 (–0·07–0·09), p=0·90	–0·011 (–0·12–0·10), p=0·83
	Adjusted model 2†	–0·001 (–0·08–0·09), p=0·99	–0·018 (–0·13–0·09), p=0·74
	Adjusted model 3‡	–0·003 (–0·06–0.05), p=0·92	0·019 (–0·05–0·09), p=0·58
	Adjusted model 4§	0·001 (–0·06–0·06), p=0·99	0·025 (–0·04–0·09), p=0·47

Models tested the associations between suspected or definite (*vs* none) psychotic experiences at age 13 years and disordered eating behaviours and body-mass index (BMI) at age 18 years. Data presented are odds ratios (95% CI) from complete case analyses (sample B) and analyses done on a sample of children for whom we had with complete exposure data and imputed confounders and outcomes (sample A, main analyses).

* Adjusted model 1: adjusted for maternal: age, marital status, education, social class, depression; and child's sex.

† Adjusted model 2: adjusted model 1 plus autistic traits at age 7 years.

‡ Adjusted model 3: adjusted model 2 plus BMI at age 13 years.

§ Adjusted model 4: adjusted model 3 plus depressive symptoms at age 13 years.

¶ Data given for this variable are coefficients (95% CI).

When we looked at individual behaviours, psychotic experiences at 13 years were associated with greater odds of binge eating at 18 years in the crude model (table 2). This association was slightly attenuated by the inclusion of child and maternal confounders; however, after adjustment for baseline depressive symptoms there was no evidence of an association with binge eating. In univariable models, psychotic experiences at 13 years were associated with purging behaviours at age 18 years (table 2). This association decreased slightly after inclusion of child and maternal confounders, and there was no association after inclusion of depressive symptoms at baseline. Psychotic episodes were strongly associated with increased risk of fasting in the univariable model, and multivariable models adjusting for child and maternal confounders and child's depressive symptoms at baseline (table 2). We showed no association between psychotic experiences and excessive exercise or BMI in any of the models.

Between 1898 and 2957 adolescents had complete data on all variables included across different outcomes (table 2), and between 2532 and 4035 adolescents had complete data on exposure and outcome (appendix). For all of our outcomes, the odds ratio and 95% CIs were comparable in magnitude across the three data samples. Complete case analyses seemed to overestimate slightly the association between psychotic experiences and purging behaviours and any disordered eating. Outcomes which were more prevalent (ie, fasting and any disordered eating) yielded strong associations with psychotic experiences at 13 years, despite comparable effect sizes with individual behaviours such as bingeing and purging showing no or weak associations with the exposure.

## Discussion

At age 18 years, a third of adolescents who had psychotic experiences at age 13 years reported some disordered eating behaviours. Compared with those who had not had psychotic experiences, children with psychotic experiences at age 13 years had 1·5 times the risk of reporting any disordered eating behaviours in late adolescence. They were also more likely to report more disordered-eating behaviours.

In line with our hypotheses, we noted that psychotic experiences at age 13 years were prospectively associated with binge eating, fasting, and purging. Some associations were weaker, or absent in the final model, possibly because of low statistical power. We did not find evidence of an association between psychotic experiences and greater excessive exercise and BMI at age 18 years. The slight overestimation of the association between psychotic experiences and purging behaviours and any disordered eating from complete case analyses could have been due to sampling biases or small sample size, resulting in greater uncertainty around study estimates. The small sample size could also have affected the strength of some associations.

The use of a large and well characterised general population cohort of UK adolescents brought several strengths to this study. First, data were collected prospectively using well validated questions, which have been extensively used in the previous studies.^19^, ^21^, ^36^ We were able to investigate temporality of associations between exposure, outcomes, and confounders—a necessary precondition for causal inference. Measuring self-reported disordered eating behaviours in the general population also has the advantage of minimising selection biases associated with the use of clinical cases (because only a few individuals with eating disorders reach clinical services),^40^ as well as capturing more common phenotypical presentations of otherwise rare clinical disorders. Finally, our dataset covered a substantial amount of the adolescent period, during which most disordered eating behaviours tend to emerge.^8^

Despite these strengths, our study also had several limitations. Although disordered-eating behaviours are more common than diagnoses, only a few adolescents reported these outcomes in our dataset. This low number of reports could have resulted in low statistical power and increased possibility of types I and II errors, especially in complete case analyses and when investigating individual behaviours and particularly when running multiple statistical tests. We saw similar effect sizes, but more precise CIs when we pooled all disordered eating behaviours together (increasing our statistical power). This finding suggests that the weak associations observed for individual outcomes could indeed have resulted from low power.

Our disordered eating definitions were broad. Some adolescents included in our outcome definition might therefore have had very low threshold behaviours (ie, occurring less frequently than that required by diagnostic manuals for a diagnosis of an eating disorder), which could have artificially diluted associations. To overcome this limitation and to investigate the association between psychotic experiences and more severe eating-disorder psychopathology, we created a variable indicating the number of co-occurring disordered-eating behaviours. We noted that children with psychotic experiences also reported more severe disordered eating outcomes. Our findings therefore appear to be consistent with the possibility that, like other disorders, eating disorders exist on a continuum of severity from disordered eating to diagnosed-eating disorders.

It is possible that losses to follow up could have resulted in some degree of selection bias. To account for this bias, we imputed missing covariate and outcome data, and with the exception of purging behaviours (for which we observed stronger associations in complete case analyses), the results were similar. Substance use could have been a potential confounder of the association between psychotic experiences and disordered eating. However, substance use before age 13 years was rare in our sample, so we did not have enough power to control for it; future studies should aim to include these behaviours.

Our measure of BMI did not account for pubertal status (as acknowledged by the researchers who developed this methodology).^32^ We did not adjust for pubertal timing at baseline because we hypothesised in our direct acyclical graph that it would exert its effect on the outcome via BMI and depressive symptoms at baseline (appendix). However, this assumption relied on our hypothesised risk mechanisms and might have missed other biological and psychological mechanisms, which should be investigated in future studies.

Finally, we were not able to measure excessive concerns with healthy eating behaviours, which might be a marker of orthorexia nervosa, because these outcomes are not measured by the Youth Risk Behaviour Surveillance System questions included in ALSPAC. Given increasing public health concerns around these behaviours, future studies should also aim to collect this information. We noted that children reporting psychotic experiences at age 13 years were more likely to have any disordered eating behaviours in adolescence with more severe symptoms, defined as a greater number of behaviours. These results align well with those from previous investigations showing that adolescents and adults with psychotic experiences are more likely to also report co-occurring disordered eating behaviours^3^ and develop diagnosed eating disorders,^11^ and those with eating disorders are more likely to have^12^ or subsequently report psychotic symptoms.^11^ However, these studies—given their cross-sectional^4^, ^12^ or retrospective nature^11^—could not clearly establish the temporality of these associations^4^, ^12^ or might have been affected by recall bias.^11^ The prospective nature of our investigation, on the other hand, allowed us to show for the first time that psychotic experiences are likely to precede the onset of disordered-eating behaviours, especially since disordered-eating behaviours tend to be rare before age 13 years and only typically emerge in mid-to-late adolescence.^8^

Similar to results from a previous study of adults that showed that those with psychotic symptoms had greater odds of subsequently developing bulimia nervosa,^11^ we noted that adolescents who reported psychotic experiences at age 13 years had greater odds in both crude models and adjusted models 1 to 3 of reporting purging and binge eating behaviours at age 18 years—core symptoms of bulimia nervosa. Binge eating is also a core symptom of binge-eating disorder for which a lifetime comorbidity with psychotic symptoms also occurs.^11^ Fasting is a restrictive behaviour which, although typical of anorexia nervosa, might also represent a non-purging compensatory behaviour in bulimia nervosa, and has been shown to be a strong predictor of onset of bingeing and purging behaviours in adolescence.^41^ Crossover between diagnoses of eating disorders is common and estimated to occur in up to 50% of cases between anorexia nervosa and bulimia nervosa.^42^ Hence, it is perhaps unsurprising that we observed an effect across the whole eating disorders behavioural spectrum as opposed to finding specific associations.

Eating disorders, particularly bulimia nervosa and binge eating disorder,^8^ are highly comorbid with mood disorders. In our analyses, adjustment for depressive symptoms attenuated the magnitude of the association between psychotic experiences and disordered eating and in some cases—eg, binge eating—completely accounted for the association. This finding suggests that transdiagnostic psychopathologies in early adolescence could increase future disordered eating behaviours. A study in this sample^7^ has shown that depressive and anxiety symptoms, and psychotic experiences occur on a continuum of severity with psychotic experiences denoting more acute psychopathological presentations rather than being distinct diagnostic entities. Psychotic experiences could therefore represent one set of early markers of more severe mental illness with transdiagnostic effects that extend, among other conditions,^3^, ^5^, ^6^ to eating disorders. The presence of shared risk factors between depression, psychosis, and eating disorders—such as bullying^43^, ^44^ or trauma^45^, ^46^—seems to corroborate this hypothesis.

Adjustment for depressive symptoms attenuated, but did not completely explain the association between psychotic experiences and purging, fasting, and any or more severe disordered eating. It is therefore possible that the association between psychotic experiences and eating disorders could also be underpinned by specific shared risk factors, such as cognitive impairments. For instance, individuals with either eating disorders or schizophrenia show similar deficits in visuospatial and global or local processing, with greater bias towards detail processing.^47^, ^48^ Most research into eating disorders has been done in clinical samples, which makes it difficult to disentangle whether cognitive deficits precede or are a consequence of eating disorders. Our finding that psychotic experiences, previously associated with several cognitive abnormalities,^19^, ^22^ are longitudinally associated with disordered eating suggests that certain cognitive impairments could precede the development of eating disorders.

Clinical accounts of eating disorders also lend plausibility to the hypothesis of commonalities across psychotic and eating disorders. For instance, it has been suggested that for a subgroup of people with concerns about their weight and shape, such symptoms could resemble delusions typically observed in psychosis.^49^ Individuals with eating disorders also recount experiences of hearing an inner voice,^50^ which can become progressively more hostile, controlling, and judgmental of their eating behaviours, weight, and shape. Unlike psychosis, however, these occurrences are not commonly understood as external voices.^51^ More longitudinal research exploring these putative shared risk mechanisms is therefore warranted.^47^

Findings from genetic studies also support the hypothesis that psychotic and eating disorders could have shared causal pathways. Several investigations using the results of large genome-wide association studies have uncovered a positive genetic correlation between schizophrenia and anorexia nervosa, and a negative correlation between both disorders and BMI.^52^, ^53^ These results suggest that schizophrenia and anorexia nervosa might be characterised by shared metabolic and developmental risk pathways. For instance, in the ALSPAC cohort, polygenic risk scores for schizophrenia have been shown to be associated with deficits in social communication,^54^ also proposed as a risk factor for eating disorders, as well as anxiety.^8^, ^55^, ^56^

There were no associations between psychotic experiences and BMI in our study. There is evidence that underweight BMI is a risk factor for schizophrenia.^57^ However, we observed that psychotic experiences were associated with greater binge-eating behaviours, shown to predict greater BMI in this population,^58^ and this finding requires further investigation. Greater binge eating behaviours in children with psychotic experiences, however, could explain the increased rates of metabolic disorder observed in drug-naive individuals with psychosis, since binge eating has been proposed as a risk factor for metabolic abnormalities independently of obesity.^59^ In our sample, psychotic experiences at 18 years have also recently been linked to increased rates of insulin resistance,^60^ also occurring in individuals who binge eat^61^ and a feature of diabetes, often comorbid with eating disorders.^62^ More research exploring these shared metabolic risk pathways is therefore warranted.

In conclusion, we showed that psychotic experiences in early adolescence were longitudinally associated with disordered eating behaviours at age 18 years, potentially with more severe presentations. Our findings provide further evidence that psychotic experiences could represent non-specific markers of adverse psychopathology in adolescence^1^, ^11^ and extend their relevance to disordered eating behaviours, previously largely overlooked by the scientific literature. Our study, in the context of broader clinical and genetic studies, supports the hypothesis of shared causal pathways between psychotic illness and eating disorders, which requires further investigation. Finally, our results suggest that increased disordered behaviours, especially cycles of binge-eating and fasting, could partly account for increased rates of metabolic abnormalities in individuals with psychotic illness,^63^ although more research investigating these mechanisms is needed.

For more on **ALSPAC** see http://www.bris.ac.uk/alspac/researchers/data-access/data-dictionary/
