# Supplementary material for: Longitudinal associations between psychotic experiences and disordered eating behaviours in adolescence: a UK population-based study
Source: Lancet Child Adolesc Health. 2018 Aug;2(8):591–9. doi: 10.1016/S2352-4642(18)30180-9 (PMC6054050; doi:10.1016/S2352-4642(18)30180-9)
Supplement: Supplementary appendix [file mmc1.pdf]

THE LANCET
Child & Adolescent Health

Supplementary appendix

This appendix formed part of the original submission and has been peer reviewed. We post it as supplied by the authors.

Supplement to: Solmi F, Melamed D, Lewis G, Kirkbride JB. Longitudinal associations between psychotic experiences and disordered eating behaviours in adolescence: a UK population-based study. *Lancet Child Adolesc Health* 2018; published online June 21. http://dx.doi.org/10.1016/S2352-4642(18)30180-9.

Web Extra Material

Table S1: Questions used to derive disordered eating behaviours outcomes

Questions	Answers	Coding	Final variable
**Binge eating**			
Sometimes people will go on an “eating binge”, where they eat an amount of food that most people would consider an unusually large amount of food (for example a large tub of ice cream or a whole packet of biscuits (containing more than 10 biscuits) or more than 3 small packets of crisps or a whole large pack of crispts), in a short period of time. During the past year, how often did you go on an eating binge	**1. Less than once a month**<br>**2. 1-3 times a month**<br>**3. once a week**<br>**4. more than once a week**<br>5. never	**Overeating**<br>5 = 0 “no”<br>**1-4 =1 “yes”**	**Binge eating**<br><br>“overeating”<br>AND<br>“loss of control”
Did you feel out of control, like you couldn’t stop eating even if you wanted to stop?	**1. Yes, usually**<br>2. Yes, sometimes<br>3. No	**Loss of control**<br>2-3 = 0 “no”<br>**1 = 1 “yes”**	
**Purging**			
During the past year, how often did you make yourself throw up (vomit) to lose weight or avoid gaining weight?	1. never<br>**2. Less than once a month**<br>**3. 1-3 times a month**<br>**4. once a week**<br>**5. 2-6 times a week**<br>**6. every day**	**Self-induced vomiting**<br>1=0 “no”<br><br>2-6 = “yes”	**Purging**<br><br>“self-induced vomiting”<br><br>OR<br><br>“use of laxatives or other medications”
During the past year, did you take laxatives or any other tablets or medicines (diet pills or water tablets) to lose weight or avoid gaining weight?	**1. yes, laxative**<br>**2. yes, other**<br>3. never	**Use of laxatives or other medications**<br><br>3 = 0 “no”<br>1-2=1 “yes”	
**Excessive exercise**			
[during the past year] did you exercise in order to lose weight or to avoid gaining weight?	1. yes sometimes<br>**2. yes frequently**<br>3. no	**Exercise for weight loss**<br>1, 3 (+children who do not exercise at all) =0 “no”<br>2 = 1 “yes”	**Excessive exercise**<br><br>Exercise for weight loss<br>AND<br><br>Guilty<br>OR<br>Difficulty to concentrate
Do you feel guilty after missing an exercise session?	1. no<br>2. yes sometimes<br>**3. yes frequently**<br>**4. do not miss any sessions**	**Guilty**<br>1-2 = 0 “no”<br>3-4= 1 “yes”	
Was it difficult to do your work or college/university work because of the amount of time you were exercising?	1. yes sometimes<br>**2. yes frequently**<br>3. no	**Difficulty to concentrate**<br>1, 3 = 0 “no”<br>2 = 1 “yes”	
**Fasting**	**Answers**	**Coding**	
During the past year, how often did you fast (not eat for at least a day) to lose weight or avoid gaining weight?	1. never<br>2. less than once a month<br>3. 1-3 times a month<br>4. once a week<br>5. two or more times a week	**Fasting**<br>1 = 0 “no”<br>2-5 = 1 “yes”	

Figure S1: Direct Acyclic Graph (DAG) showing the hypothesised relationship between pubertal status, BMI and depression in relation to the association between psychotic experiences and disordered eating

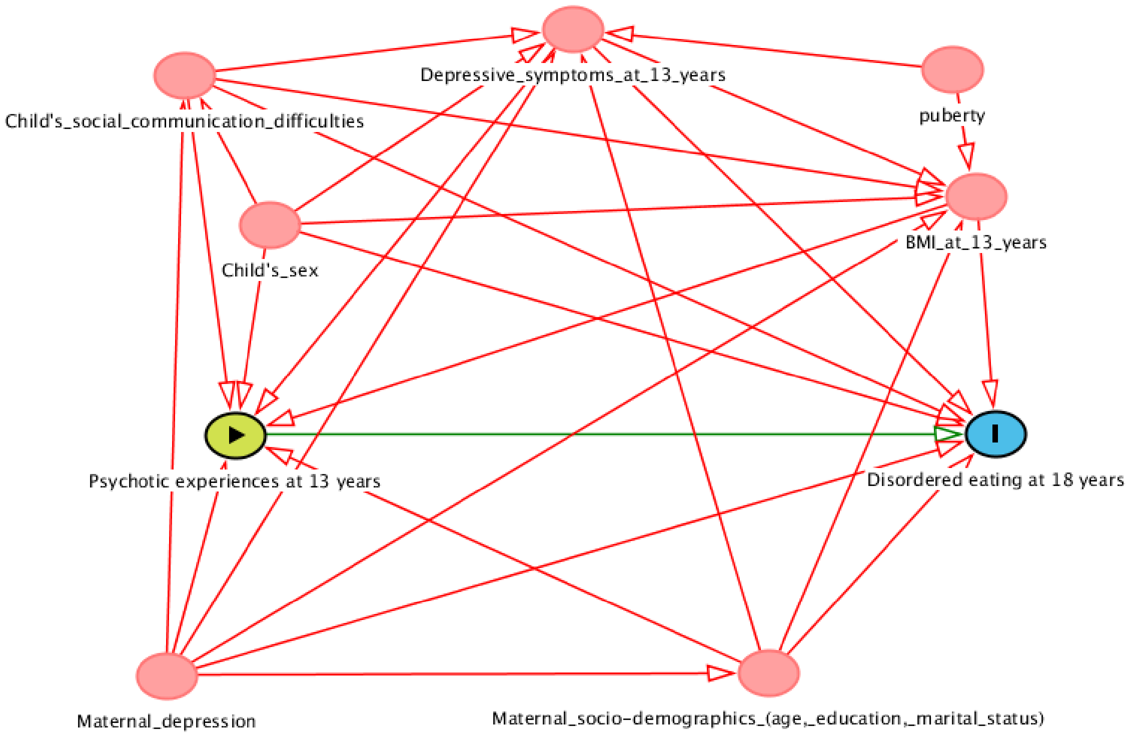

In this DAG (modelling our causal assumptions between variables included in our model and developed using the online tool available at http://www.dagitty.net/) the green and blue circles indicate our study exposure and outcome, respectively. Green arrows indicate causal paths, whilst red arrows indicate biasing paths, which should be accounted for in the analyses.

Child's sex was included as a potential confounder as it has previously shown that in this sample, both PEs and disordered eating is more prevalent in girls[1,2]. Maternal characteristics (age, marital status, highest education) were included as proxy of socio-economic position also associated with both PEs and disordered eating and BMI.[3,4]d Maternal and child's depressive symptoms, autistic traits, BMI were included as they have been posited to be risk factors both childhood psychopathology (including eating disorders and psychotic symptoms) and BMI. [5–10]

As shown in this DAG, minimal sufficient adjustment for estimating the total effect of Psychotic Experiences at 13 years on Disordered eating at 18 years required the inclusion of the following confounding variables: maternal depression and socio-demographic characteristics (age, marital status, and education); as well as child's: sex, social communication difficulties at age 7 years, and BMI and depressive symptoms at age 13 years. Pubertal status influences disordered eating via its effect on depressive symptoms and BMI, with no direct effect on disordered eating at age 18 years. Hence, it was not adjusted for in the model.

Table S2: Predictors of outcome missingness among participants with complete exposure

	**Missing Outcome data N = 1,888 (29·7%) OR (95%CI)**
Psychotic experiences *(ref No)*	
Yes	1·08 (0·91 – 1·27)
Sex *(ref Male)*	
Female	0·50 (0·45 – 0·56)**
Maternal marital Status *(ref never married)*	
Ever Married	0·75 (0·65 – 0·88)**
Widowed, Divorced, separated	1·05 (0·80 – 1·39)
Maternal highest academic qualification *(ref up to A level)*	
Degree or Higher	0·37 (0·30 – 0·44)**
Child's Depressive symptoms	0·99 (0·98 – 1·00)
Autistic traits	1·04 (1·02 - 1·06)**
Maternal depressive symptoms	1·03 (1·02 – 1·04)**
Maternal age	0·94 (0·93 – 0·96)**
Child's BMI at 13 years	1·06 (1·00 – 1·12)**

** p≤0·05

*0·1>p>0·05

Table S3: Logistic, linear (for BMI), and negative binomial (for number of disordered eating behaviours) regression models testing the associations between suspected or definite psychotic experiences and disordered eating behaviours and Body Mass Index at age 18. The sample includes children for whom we had with complete exposure and outcome data, and imputed confounders (referred to as sample C in the manuscript)

	**Exposure: psychotic experiences at age 13 years (suspected or definite vs. none)**
	**Complete exposure and outcome, imputed confounders (Sample C) OR (95%CI)**
**Binge eating**	*N = 2,581*
Crude model	2·16 (1·30, 3·57), p=0·0028
Adjusted model 1[a]	1·95 (1·16, 3·28), p=0·011
Adjusted model 2[b]	1·95 (1·16, 3·28), p=0·011
Adjusted model 3[c]	1·96 (1·16, 3·30), p=0·012
Adjusted model 4[d]	1·53 (0.89, 2·62), p=0·13
**Purging**	*N = 2,564*
Crude model	1·88 (1·21, 2·93), p=0·0050
Adjusted model 1[a]	1·79 (1·13, 2·83), p=0.012
Adjusted model 2[b]	1·79 (1·13, 2·83), p=0.013
Adjusted model 3[c]	1·79 (1·13, 2·83), p=0.013
Adjusted model 4[d]	1·60 (1·00, 2·57), p=0·053
**Excessive exercise**	*N = 2,573*
Crude model	0·75 (0.36, 1.58), p=0.45
Adjusted model 1[a]	0·73 (0.35, 1.53), p=0.40
Adjusted model 2[b]	0·73 (0.34, 1.53), p=0.40
Adjusted model 3[c]	0·72 (0.34, 1.52), p=0.39
Adjusted model 4[d]	0·65 (0.31, 1.40), p=0.27
**Fasting**	*N = 2,588*
Crude model	2·13 (1·53, 2·98), p<0·0001
Adjusted model 1[a]	2·06 (1·45, 2·93), p<0·0001
Adjusted model 2[b]	2·06 (1·45, 2·93), p<0·0001
Adjusted model 3[c]	2·06 (1·45, 2·94), p<0·0001
Adjusted model 4[d]	1·67 (1·16, 2·41), p=0·0059

Table S3 (continued)

	Exposure: psychotic experiences at age 13 years (suspected or definite vs. none)
	OR (95%CI)
**Any disordered eating behaviour**	***N = 2,532***
Crude model	2·03 (1·51, 2·72), p<0·0001
Adjusted model 1[a]	2·01 (1·47, 2·74), p<0·0001
Adjusted model 2[b]	2·00 (1·47, 2·73), p<0·0001
Adjusted model 3[c]	2·01 (1·47, 2·75), p<0·0001
Adjusted model 4[d]	1·66 (1·20, 2·30), p=0·002
	**Coefficient (95%CI)**
**Number of disordered eating behaviour**	**N = 2,532**
Crude model	0·58 (0·28, 0·87), p<0·0001
Adjusted model 1[a]	0·50 (0·22, 0·78), p<0·0001
Adjusted model 2[b]	0·51 (0·23, 0·79), p<0·0001
Adjusted model 3[c]	0·52 (0·24, 0·80) p<0·0001
Adjusted model 4[d]	0·38 (0·10, 0·66), p=0·0076
**BMI**	*N = 4,035*
Crude model	0·077 (-0·09, 0·10), p=0·87
Adjusted model 1[a]	-0·017 (-0·11, 0·08), p=0·72
Adjusted model 2[b]	-0·023 (-0·12, 0·07), p=0·62
Adjusted model 3[c]	-0·007 (-0·07, 0·05), p=0·82
Adjusted model 4[d]	-0·003 (-0·07, 0·06), p=0·91

REFERENCES

1 Solmi F, Colman I, Weeks M, Lewis G, Kirkbride JB. Trajectories of Neighborhood Cohesion in Childhood, and Psychotic and Depressive Symptoms at Age 13 and 18 Years. *J Am Acad Child Adolesc Psychiatry* 2017.

2 Micali N, Solmi F, Horton NJ, Crosby RD, Eddy KT, Calzo JP, *et al.* Adolescent Eating Disorders Predict Psychiatric, High-Risk Behaviors and Weight Outcomes in Young Adulthood. *J Am Acad Child Adolesc Psychiatry* 2015; **54**: 652–659.e1.

3 Drukker M, Krabbendam L, Driessen G, van Os J. Social disadvantage and schizophrenia. *Soc Psychiatry Psychiatr Epidemiol* 2006; **41**: 595–604.

4 Larsen PS, Strandberg-Larsen K, Olsen EM, Micali N, Nybo Andersen A-M. Parental characteristics in association with disordered eating in 11- to 12-year-olds: A study within the Danish National Birth Cohort. *Eur Eat Disord Rev* 2018. doi:10.1002/erv.2599.

5 Reed ZE, Micali N, Bulik CM, Smith GD, Wade KH. Assessing the causal role of adiposity on disordered eating in childhood, adolescence, and adulthood: A Mendelian randomization analysis. *Am J Clin Nutr* 2017; **106**: 764–72.

6 Wahlbeck K, Forsén T, Osmond C, Barker DJP, Eriksson JG, V L, *et al.* Association of Schizophrenia With Low Maternal Body Mass Index, Small Size at Birth, and Thinness During Childhood. *Arch Gen Psychiatry* 2001; **58**: 48.

7 Baron-Cohen S, Jaffa T, Davies S, Auyeung B, Allison C, Wheelwright S. Do girls with anorexia nervosa have elevated autistic traits? *Mol Autism* 2013; **4**: 24.

8 St Pourcain B, Robinson EB, Anttila V, Sullivan BB, Maller J, Golding J, *et al.* ASD and schizophrenia show distinct developmental profiles in common genetic overlap with population-based social communication difficulties. Mol. Psychiatry. 2017. doi:10.1038/mp.2016.198.

9 Verbeek T, Bockting CLH, van Pampus MG, Ormel J, Meijer JL, Hartman CA, *et al.* Postpartum depression predicts offspring mental health problems in adolescence independently of parental lifetime psychopathology. *J Affect Disord* 2012; **136**: 948–54.

10 Lampard AM, Franckle RL, Davison KK. Maternal depression and childhood obesity: A systematic review. *Prev Med (Baltim)* 2014; **59**: 60–7.
